# Assessment of photobiomodulation combined with new restorative material for teeth with molar incisor hypomineralization on control of hypersensitivity and longevity of restorations: Protocol for a randomized controlled blind clinical trial

**DOI:** 10.1371/journal.pone.0329641

**Published:** 2025-08-13

**Authors:** Caroline Diniz Pagani Vieira Ribeiro, Amanda Rafaelly Honório Mandetta, Fabiana Car Pernomiam, Caroline Cristina Batista De Camargo, Iara Maria Freitas Romano, Ana Paula Taboada Sobral, Marcela Letícia Leal, Raquel Agnelli Mesquita-Ferrari, Kristianne Porta Santos Fernandes, Anna Carolina Ratto Tempestini Horliana, Lara Jansiski Motta, Cinthya Cosme Gutierrez Duran, Sandra Kalil Bussadori

**Affiliations:** 1 Biophotonics Medicine Postgraduate Program, Universidade Nove de Julho, São Paulo, Brazil; 2 Postgraduate Programme in Rehabilitation Sciences, Universidade Nove de Julho, São Paulo, Brazil; 3 Postgraduation Programme in Health and Environment, Universidade Metropolitana de Santos, Santos, Brazil; Indiana University School of Dentistry, UNITED STATES OF AMERICA

## Abstract

Molar incisor hypomineralization (MIH) is a qualitative defect of enamel development that occurs in the mineralization phase. MIH affects one or more permanent molars and, occasionally, permanent incisors. The aim of the proposed study is to determine whether photobiomodulation combined with a new self-cure resin improves hypersensitivity in molars with MIH (primary outcome). Secondary outcomes include assessing the clinical performance of the self-cure composite resin in terms of restoration longevity and comparing the effectiveness of three interventions—photobiomodulation combined with self-cure resin, self-cure resin alone, and photobiomodulation combined with bulkfill photopolymerizable resin—in controlling hypersensitivity over time. Permanent molars with MIH in patients 6–10 years of age will be allocated to three groups. Group 1: photobiomodulation + self-cure composite resin restoration; Group 2: self-cure composite resin restoration; Group 3: photobiomodulation + restoration in bulk-fill photopolymerizable composite resin. Photobiomodulation will be performed in a single session involving low-level laser administered to four different points. The laser will be used at a wavelength of 808 nm, power of 100 mW and energy of 1 J per points; irradiance will be 3571 mW/cm^2^, with a total radiant exposure of 35.7 J/cm^2^. Data normality will be checked using the Shapiro-Wilk test, and variance homogeneity will be assessed with the Levene test. Descriptive statistics will be used to present the data, with continuous variables expressed as mean and standard deviation, and categorical variables by relative frequency. To compare the Wong-Baker Faces Pain Rating Scale and SCASS scales, repeated measures ANOVA will be employed, considering the 3 groups and 5 time points. Bonferroni adjustment will be applied for post-hoc comparisons. Sphericity will be tested with Mauchly’s test, and if violated, Greenhouse-Geisser correction will be applied. A significance level of 0.05 will be adopted.

**Trial registration:**

ClinicalTrials.gov NCT06538142

## Introduction

Molar-Incisor Hypomineralization (MIH) is a developmental enamel defect that impairs mineralization during the amelogenesis process, primarily affecting the first permanent molars and, in some cases, the incisors [1]. Molar-Incisor Hypomineralization (MIH) is characterized by well-defined, asymmetrical opacities with varying color shades. Darker opacities exhibit a higher susceptibility to fractures due to a greater compromise in their mineral composition [1]. MIH poses a significant clinical challenge due to dental hypersensitivity, an increased risk of caries, and difficulties in achieving adequate adhesion of restorative materials [2].

Hypersensitivity is a common symptom in patients with MIH and may occur in teeth with or without post-eruptive fractures. The severity of clinical manifestations is directly correlated with the intensity of hypersensitivity [3]. Although the underlying mechanisms of hypersensitivity in MIH-affected teeth are not yet fully elucidated, it is hypothesized that chronic stimuli contribute to a subclinical pulpal inflammation. This process is likely driven by bacterial infiltration through the porous enamel, leading to persistent nociceptive responses [4].

Photobiomodulation with low- or high-power laser is a potential treatment method for dentinal hypersensitivity [5]. Photobiomodulation (PBM) with low-level laser (LLL), such as GaAlAs or He-Ne, has no known side effects when administered with the appropriate settings. The mechanism of action is based on the induction of changes in nerve transmission to control pain. Nerve cells are stimulated and the sodium/potassium pump in the cell membrane increases the amplitude of the action potential, thus blocking the transmission of the pain stimulus. Moreover, PBM enhances cell regeneration capacity and reduces inflammation [6,7].

Teeth affected by Molar-Incisor Hypomineralization (MIH) exhibit a reduction in mineral content, accompanied by an increase in organic content due to the retention of proteins during the mineralization process. This structural modification compromises the adhesion of restorative materials to the hypomineralized enamel, making it one of the main factors associated with the failure of restorative procedures. As a result, there is a higher failure rate in treatments, often requiring re-treatments and restorative replacements over time. [8,9].

The preferred approach for teeth with MIH and post-eruptive fractures is restorative treatment with or without absolute isolation. Relative isolation has been shown to be effective in MIH teeth restored with hybrid glass materials [10]. Moreover, some studies have not observed significant differences in the longevity of resin restorations when comparing relative and absolute isolation methods [10–13].

Stela composite resin (Southern Dental Industries) is an innovative, high-performance, self-cure composite with a catalyst that initiates the curing process at the restoration interface. The polymerization sequence attenuates contraction tension by enabling an interface with no gaps, reducing postoperative sensitivity and the risk of premature failure [14–19]. This product has a simple process and fast execution, which is particularly beneficial for children with MIH. There is a need for the investigation of procedures aimed at reducing hypersensitivity and materials that can enhance the longevity of restorations, especially on teeth affected by MIH to improve clinical outcomes, minimize the need for additional interventions and enhance the quality of life of patients.

Therefore, the aim of the proposed study is to determine whether photobiomodulation combined with a new photopolymerizable resin improves hypersensitivity in MIH-affected carious molars. The secondary objectives are to evaluate the effectiveness in controlling hypersensitivity over time and to assess the clinical performance of the self-cure composite resin in terms of restoration longevity.

## Methods

### Study design

**Fig 1:**
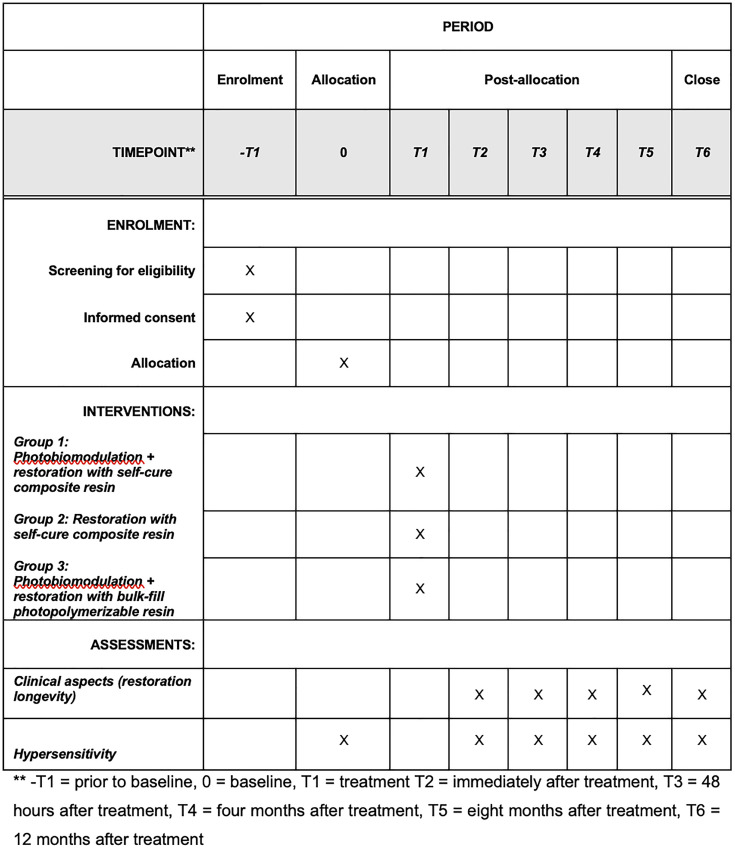
Timeframe of enrolment, intervention and assessments.

A randomized, controlled, single-blinded clinical trial will be conducted. The present protocol study followed the Standard Protocol Items for Randomized Trials (SPIRIT statement), as presented in Fig 1 and S1 File (SPIRIT checklist). The recruitment and data collection period will take place between February 1, 2025, and January 1, 2026. The results are expected to be available by August 2026. The clinical trial protocol is provided as Supporting Information file (S2 File).

### Ethical aspects

The study will be conducted in accordance with the ethical precepts stipulated in the Declaration of Helsinki (World Medical Association, 2008) and the norms governing research involving human beings stipulated in Resolutions nº 466/12 and 510/2016 of the National Board of Health. The study received approval from the Human Research Ethics Committee of Nove de Julho University (certificate number: 83027624.2.0000.5511/approval date: December 4, 2024) and was registered in ClinicalTrials.gov (NCT06538142, Last Update: January 17, 2025). The legal guardians will agree to the children’s participation by signing a statement of informed consent. Recruitment will begin after approval from the Human Research Ethics Committee. The participants will be informed that they can withdraw from the study at any time for any reason, if they so desire. The researchers may withdraw participants from the study if it is considered necessary.

### Objectives

Primary Objective: To determine whether photobiomodulation combined with a new photopolymerizable resin improves hypersensitivity in MIH-affected carious molars.

Secondary objectives:

- To compare the effectiveness of three interventions: photobiomodulation combined with self-cure resin, self-cure resin alone, and photobiomodulation combined with bulkfill photopolymerizable resin in controlling hypersensitivity over time.
- To assess the clinical performance of the self-cure composite resin in terms of restoration longevity.

### Description of sample

Teeth will be selected from male and female children 6–10 years of age with no distinction as to ethnicity enrolled for outpatient treatment at the Aria College in the city of Brasília with first molars that have MIH and restorative treatment need. The study has received a letter of approval from Aria College, granting permission for the research to be conducted in the Department of Pediatric Dentistry at their facility. Individuals who met the eligibility criteria will immediately be randomized and treated. Each participant will receive treatment on a single molar affected by MIH. In cases where more than one tooth meets the eligibility criteria, the tooth to be included in the study will be selected randomly through a simple lottery method. This selection process will occur prior to the randomization of the interventions to ensure a clear and unbiased allocation of treatments. The trial will be reported following the CONSORT guidelines.

### Inclusion criteria

- Male and female children;
- 6–10 years of age;
- At least one permanent first molar with MIH (codes 5 and 6 of molar-incisor hypomineralization severity scoring system (MIH-SSS) [20]
- Presence of hypersensitivity
- Direct viewing and access.

### Exclusion criteria

- Clinically: signs or symptoms of pulp involvement;
- Radiographically: evidence of pulp involvement (initial periapical radiograph);
- Teeth with MIH with no indication for direct restorative treatment (multiple surfaces associated with large extensions) [21].
- Partially erupted teeth;
- Previous restorative treatment.
- Patients who have undergone previous desensitizing treatments

### Involvement of patient and public

The guardians of the patients will not be involved in the design of the study. After data analysis, the guardians will have the opportunity to participate in a meeting during which the results will be shared. The statement of informed consent signed by the guardians of the study will explain that confidentiality of the data will be ensured.

### Sample size calculation

The sample size will be estimated using the G*Power software (version 3.1, Franz Faul, Unikiel, Germany) [22] for a priori repeated measures ANOVA. An effect size of 0.352, derived from the group mean dispersions reported in the literature [23], will be considered. The alpha error will be set at 0.05, statistical power at 80%, and the design includes 3 groups and 5 repeated measures. The final calculation indicates the need for 66 individuals in total, corresponding to 22 participants per group. To increase the robustness of the results and compensate for potential dropouts, the sample will be inflated by 15%, resulting in 25 individuals per group and a total of 75 participants.

### Randomization

The participants will be randomly allocated to the different groups using block randomization. A computer-generated sequence (random.org; Randomness and Integrity Services, Dublin, Leinster, Ireland) will be used to allocate each participant to a specific group maintaining a 1:1:1 proportion (equal number of participants in all groups). Randomization will be performed in 5 blocks, with 15 participants per block, totaling 75 participants across the study. Allocation concealment will be ensured with the use of sequentially numbered sealed opaque envelopes.

Teeth selected for treatment will be randomized into three groups:

**Group 1**: Photobiomodulation + restoration with self-cure composite resin

**Group 2**: Restoration with self-cure composite resin

**Group 3**: Photobiomodulation + restoration with photopolymerizable bulk-fill composite resin

### Blinding

All treatments across the three groups will be carried out by a single operator, who will have completed training and calibration sessions during the initial phase of the study. Clinical assessments after 48 hours as well as four, eight and twelve months after treatment (follow-up) will be performed by an examiner blinded to allocation (single-blind study). The operator and examiner will undergo previous calibration exercises, evaluating approximately 20 cases, with the calculation of the Kappa statistic to ensure greater than 85% inter-observer agreement. The calibration will follow the criteria of the modified index of the United States Public Health Service (USPHS) [24,25] and the MIH-SSS diagnostic criteria [20]. The data will be analyzed by a blinded statistician.

### Interventions

#### Photobiomodulation.

Photobiomodulation will be performed with a low-power infrared diode laser (DMC, São Carlos, Brazil) in a single session. The laser will be administered at three points in contact and perpendicular to the surface on the vestibular mesial and distal cervical thirds as well as the center of the occlusal face [26]. Each point will be irradiated for 30 seconds with an energy of 3 J. The photobiomodulation parameters are described in Table 1.

**Table 1:** Photobiomodulation parameters.

Photobiomodulation parameters	
Application method	Contact
Number of points irradiated	3
Number of sessions	1
Wavelength (nm)	808 ± 10
Irradiance (mW/cm^2^)	3571
Spectral width (FWHM)	4.8 ± 2 nm
Operating mode	Continuous
Power	100 mW
Beam type	Multi-mode
Beam area [cm^2^]	0.028
Total exposure time	30 s
Radiant exposure [J/cm^2^]	35.7
Energy per point [J]	1
Total energy [J]	3

### Clinical protocol

#### Group 1 – photobiomodulation + restoration with self-cure composite resin.

1. Initial periapical radiograph;
2. Hypersensitivity reading (Wong-Baker Faces Pain Rating Scale for subjective pain of volunteer and SCASS for examiner’s assessment);
3. Application of photobiomodulation;
4. Relative isolation (lip bumper, cotton roll and aspirator);
5. Selective removal of carious tissue with excavator, which involves complete removal of carious dentin from the surrounding walls followed by partial removal of softened dentin from the pulp wall (only Code 6 of MIH-SSS criteria);
6. Cleaning with cotton and water;
7. Application of primer (Stela; SDI, Melbourne, Vic, Australia) on dentin and/or enamel, wait 5 seconds;
8. Application of mild compressed air on adhesive for 3 seconds;
9. Restoration with self-cure composite resin (Stela; SDI, Melbourne, Vic, Australia) extending to adjacent demarcated opacities;
10. Clinical follow up after 48 hours and at four-month intervals for a period of 12 months (modified USPHS index);
11. Hypersensitivity reading after 48 hours and at four-month intervals for a period of 12 months (Wong-Baker Faces Pain Rating Scale for subjective pain of volunteer and SCASS for examiner’s assessment).

#### Group 2 – restoration with self-cure composite resin.

Same as previous sequence (Group 1) with exception of Item 3.

#### Group 3 – photobiomodulation + restoration with bulk-fill composite resin.

1. Initial periapical radiograph;
2. Hypersensitivity reading (Wong-Baker Faces Pain Rating Scale for subjective pain of volunteer and SCASS for examiner’s assessment;
3. Relative isolation (lip bumper, cotton roll and aspirator);
4. Application of photobiomodulation;
5. Selective removal of carious tissue with excavator, which involves complete removal of carious dentin from the surrounding walls followed by partial removal of softened dentin from the pulp wall (only Code 6 of MIH-SSS criteria);
6. Selective etching of enamel adjacent to demarcated opacities with 35% phosphoric acid (Ultra Etch; Ultradent, Indaiatuba, SP, Brazil) for 20 seconds;
7. Active application of universal adhesive (Ambar; FGM, Joinville, SC, Brazil) on dentin and enamel for 20 seconds (repeat procedure);
8. Application of mild compressed air on adhesive for 5 seconds;
9. Photoactivation for 10 seconds, peak of 1200 Mw/cm^2^ (Radii Cal; SDI, Melbourne, Vic, Australia);
10. Restoration with Tetric N Ceram bulk-fill composite resin (Tetric N Ceram Bulk Fill; Ivoclar Vivadent, Barueri, SP, Brazil) with increments up to 4 mm, extending to demarcated opacities;
11. Photoactivation for 20 seconds (Radii Cal; SDI, Melbourne, Vic, Australia);
12. Clinical follow up after 48 hours and at four-month intervals for a period of 12 months (modified USPHS index);
13. Hypersensitivity reading after 48 hours and at four-month intervals for a period of 12 months (Wong-Baker Faces Pain Rating Scale for subjective pain of volunteer and SCASS for examiner’s assessment).

The interventions will begin without an initial application of local anesthesia. The children will be informed that anesthesia is available and can be administered at any time during the procedure, if necessary.

A children’s toothpaste with 1100 ppm fluoride, which does not contain any components specifically designed for treating dentin hypersensitivity, will be provided to all participants. This toothpaste will be used for daily oral hygiene during the study to ensure consistency across all participants and avoid any confounding factors related to hypersensitivity treatment.

### Assessments

#### Radiographic assessments.

Initial periapical radiographs will be obtained to discard the possibility of pulp involvement. Follow-up radiographs will be taken in cases of pain symptoms that justify additional exposure.

#### Clinical assessment.

The clinical assessment will be performed by an examiner blinded to the allocation of the teeth to the different groups. The criteria will be the retention of the restorative material in the cavity, posteruptive breakdown of the enamel adjacent to the restoration and the occurrence of secondary caries. The criteria of the modified USPHS index will be used for the assessment. The restoration will be characterized as a failure and the tooth will be excluded from the study if the C score is determined for any of the USPHS criteria [27]. Photographs will be taken using a digital single-lens reflex (DSLR) camera (Canon EOS 700D; Canon, Tokyo, Honshu, Japan) to complement the clinical data. These photographs will be taken before and after the restorative treatment, as well as during all follow-up periods, following the same standardized parameters. This will ensure consistency in clinical data collection. The visual demonstration will contribute to any necessary clarifications and make the discussion and documentation of the cases more efficient [28].

#### Hypersensitivity assessment.

Pain perception in children was evaluated through the Wong-Baker Faces Pain Rating Scale [29] and the Schiff Cold Air Sensitivity Scale (SCASS) [30] will be used for the assessment of the operator and examiner. Following isolation of the neighboring teeth with gauze, compressed air will be applied to the tooth with MIH for three seconds. The SCASS is scored as follows: 0 (no reaction); 1 (no reaction, but patient reports discomfort); 2 (patient reacts and moves away from stimulus); and 3 (patient reacts and asks operator to stop) [3]. Hypersensitivity will be measured using the Wong-Baker Faces Pain Rating Scale and SCASS prior to the procedure, 48 hours after restorative treatment and at four-month intervals for the 12-month follow-up period.

### Statistical analysis

The data will be presented using descriptive statistics. Continuous variables will be described with mean and standard deviation, and categorical variables by relative frequency. Statistical analyses will be conducted using SPSS software (version 28.0, IBM, USA). For comparisons of the Wong-Baker Faces Pain Rating Scale and SCASS scales, a repeated measures analysis of variance (ANOVA) will be used, considering the three groups and five time points of the study. This analysis will address the objective of comparing the effectiveness of interventions in controlling hypersensitivity over time. Post-hoc comparisons will be made using the Bonferroni adjustment to control for Type I error in multiple comparisons. The normality of the data distribution and the homogeneity of variance will be confirmed by the Shapiro-Wilk and Levene tests, respectively. The presence of sphericity will be verified using the Mauchly test, and if violated, corrections such as Greenhouse-Geisser will be applied. For secondary outcomes, repeated measures ANOVA will also be used to compare differences over time among the three intervention groups. Post-hoc analyses with Bonferroni correction will be applied when necessary. Effect sizes (partial eta squared) will be reported to assess the magnitude of differences between interventions. Additionally, Pearson or Spearman correlation coefficients will be used to explore associations between changes in hypersensitivity and secondary outcomes, depending on data distribution. Sensitivity analyses using an intention-to-treat approach may also be performed to assess the robustness of the findings. A significance level of 0.05 will be adopted for all analyses.

## Discussion

Molar incisor hypomineralization exerts a negative impact on the lives of affected individuals. The search for effective conservative treatment to ensure the reduction in hypersensitivity, preservation of the tooth structure and longevity of the restoration seems to be the most adequate choice for teeth that require early treatment [28].

Teeth with MIH can remain hypersensitive even after restorative treatment. Therefore, effective pain control is indispensable for the clinical success of the restoration and improvement in the patient’s quality of life [31].

Inadequate restoration longevity in teeth with MIH is a limitation of the treatment and one of the main challenges that dentists face. Besides the additional financial expenses, the need for constant retreatment can cause emotional exhaustion in patients [32]. Thus, there is a need for a better understanding of the real effect of novel materials that provide effective long-term treatment.

## Supporting information

S1 File SPIRIT checklist. (DOCX)

S2 File Clinical trial protocol. (PDF)
