# Supplementary material for: Assessment of photobiomodulation combined with new restorative material for teeth with molar incisor hypomineralization on control of hypersensitivity and longevity of restorations: Protocol for a randomized controlled blind clinical trial
Source: PLoS One. 2025 Aug 13;20(8):e0329641. doi: 10.1371/journal.pone.0329641 (PMC12349719; doi:10.1371/journal.pone.0329641)
Supplement: S2 File — (PDF) [file pone.0329641.s002.pdf]

UNIVERSIDADE NOVE DE JULHO
PROGRAMA DE PÓS-GRADUAÇÃO MEDICINA - BIOFOTÔNICA

CAROLINE DINIZ PAGANI VIEIRA RIBEIRO

AVALIAÇÃO DA FOTOBIOMODULAÇÃO ASSOCIADA A UM NOVO MATERIAL RESTAURADOR EM DENTES COM HIPOMINERALIZAÇÃO MOLAR INCISIVO NO CONTROLE DA HIPERSENSIBILIDADE E NA LONGEVIDADE DAS RESTAURAÇÕES: ENSAIO CLÍNICO CONTROLADO RANDOMIZADO E CEGO

São Paulo
2024

CAROLINE DINIZ PAGANI VIEIRA RIBEIRO

AVALIAÇÃO DA FOTOBIOMODULAÇÃO ASSOCIADA A UM NOVO MATERIAL RESTAURADOR EM DENTES COM HIPOMINERALIZAÇÃO MOLAR INCISIVO NO CONTROLE DA HIPERSENSIBILIDADE E NA LONGEVIDADE DAS RESTAURAÇÕES: ENSAIO CLÍNICO CONTROLADO RANDOMIZADO E CEGO

Projeto de pesquisa para programa de Doutorado

Orientadora: Profa. Dra. Sandra Kalil Bussadori

Núcleo de Pesquisa: Medicina-Biofotônica

São Paulo

2024

Resumo

Hipomineralização molar incisivo (MIH) é um defeito qualitativo do desenvolvimento do esmalte que ocorre na fase de mineralização. MIH afeta um ou mais molares permanentes e, ocasionalmente, incisivos permanentes. O objetivo do estudo proposto é determinar se a fotobiomodulação combinada com uma nova resina autopolimerizável melhora a hipersensibilidade em molares com MIH (resultado primário). Os resultados secundários incluem avaliar o desempenho clínico da resina composta autopolimerizável em termos de longevidade da restauração e comparar a eficácia de três intervenções — fotobiomodulação combinada com resina autopolimerizável, resina autopolimerizável sozinha e fotobiomodulação combinada com resina fotopolimerizável bulkfill — no controle da hipersensibilidade ao longo do tempo. Molares permanentes com MIH em pacientes de oito a 12 anos de idade serão alocados em três grupos. Grupo 1: fotobiomodulação + restauração em resina composta autopolimerizável; Grupo 2: restauração em resina composta autopolimerizável; Grupo 3: fotobiomodulação + restauração em resina composta fotopolimerizável bulkfill. A fotobiomodulação será realizada em sessão única envolvendo laser de baixa potência administrado em quatro pontos diferentes. O laser será utilizado em comprimento de onda de 808 nm, potência de 100 mW e energia de 1 J por ponto; a irradiância será de 3571 mW/cm², com exposição radiante total de 35,7 J/cm². A normalidade dos dados será verificada pelo teste de Shapiro-Wilk, e a homogeneidade da variância será avaliada pelo teste de Levene. Estatística descritiva será utilizada para apresentação dos dados, com variáveis contínuas expressas em média e desvio padrão, e variáveis categóricas por frequência relativa. Para comparação das escalas VAS e SCASS, será empregada ANOVA de medidas repetidas, considerando os 3 grupos e 5 pontos no tempo. O ajuste de Bonferroni será aplicado para comparações post-hoc. A esfericidade será testada pelo teste de Mauchly e, se violada, será aplicada a correção de Greenhouse-Geisser. Será adotado um nível de significância de 0,05.

Palavras-chave: MIH, hipersensibilidade, resina, fotobiomodulação

1. Introdução

A Hipomineralização dos Molares e Incisivos (HMI) é caracterizada como um distúrbio multifatorial complexo do desenvolvimento qualitativo do esmalte (DDE), com influência genética, que impacta predominantemente os primeiros molares permanentes e, ocasionalmente, os incisivos permanentes. Clinicamente, a HMI se manifesta por meio de opacidades demarcadas que exibem uma gama de tonalidades, variando de branco cremoso a amarelo acastanhado. Essas opacidades mais escuras tendem a apresentar uma área afetada mais porosa, aumentando sua suscetibilidade a fraturas [1]. Os principais obstáculos terapêuticos decorrentes dessa condição incluem o aumento do risco de cárie, questões estéticas, hipersensibilidade dentária, dificuldade na adesão de materiais restauradores e potenciais falhas no tratamento [2].

A hipersensibilidade dentinária (HD) a estímulos térmicos e mecânicos é um sintoma recorrente em pacientes com HMI. No esmalte altamente poroso presente nessas condições, propriedades como isolamento e condutividade térmica encontram-se comprometidas. Dentes afetados por HMI, caracterizados por opacidades demarcadas e restaurações atípicas, frequentemente manifestam hipersensibilidade leve a moderada, enquanto a hipersensibilidade grave geralmente está associada a dentes com fraturas pós-eruptivas do esmalte [3].

O mecanismo da hipersensibilidade em HMI não é completamente compreendido, mas sugere-se que a alta porosidade do esmalte afetado facilite a invasão bacteriana nos túbulos dentinários, desencadeando uma inflamação pulpar subclínica [4].

Um método potencial para o tratamento da HD é a utilização de fotobiomodulação com lasers de baixa ou alta potência [5]. A fotobiomodulação (PBM) com laser de baixa potência (LLL), como o GaAlAs ou o He-Ne, quando administrada com parâmetros apropriados, não demonstra efeitos adversos conhecidos. Seu mecanismo de ação baseia-se na indução de alterações na transmissão nervosa para controlar a dor. A célula nervosa é estimulada e a bomba de sódio/potássio em sua membrana aumenta a amplitude do potencial de ação, bloqueando assim a transmissão do estímulo doloroso. Além disso, o efeito do PBM através da aplicação de LLL promove irrigação e capacidade de regeneração celular, além de reduzir a inflamação [6,7].

O esmalte hipomineralizado apresenta propriedades mecânicas inferiores, demonstrando menor dureza e módulo de elasticidade. O alto teor de proteínas constitui o principal obstáculo para a adesão dos materiais restauradores a esse substrato. Consequentemente, é comum observar falhas no tratamento e a necessidade de retratamento [8,9].

O tratamento restaurador é indicado para dentes com HMI e fraturas. Apesar das opiniões divergentes na literatura quanto ao uso do isolamento relativo em procedimentos restauradores, ele tem se mostrado eficaz em dentes com HMI restaurados com materiais híbridos de vidro [10]. Além disso, alguns estudos não identificaram diferenças significativas na longevidade das restaurações de resina ao comparar os dois métodos de isolamento, seja relativo ou absoluto [10-13].

A resina composta Stela (SDI) é um compósito inovador autopolimerizável de alto desempenho que contém um catalisador que inicia o processo de cura na interface de restauração. Esta sequência de polimerização atenua a tensão de contração por permitir uma interface sem fendas, reduzindo a sensibilidade pós-operatória e o risco de falha prematura [14-19]. Ela apresenta um processo simplificado e de rápida execução, o que pode ser particularmente benéfico em crianças com HMI.

2. Justificativa

A investigação de procedimentos que visem a redução da hipersensibilidade e materiais que possam estender a vida útil das restaurações, especialmente em dentes afetados pela Hipomineralização Molar-Incisivo (HMI), é uma necessidade premente. Isso visa aperfeiçoar os resultados clínicos, minimizar a necessidade de intervenções adicionais e melhorar a qualidade de vida dos pacientes.

3. Hipótese

A nossa hipótese é que a ação neural (fotobiomodulação) combinada a restauração com uma resina autopolimerizável promoverá redução da hipersensibilidade em dentes com HMI e fratura pós-eruptiva com ou sem lesão de cárie.

4. Objetivos

4.1 **Objetivos Gerais**

Avaliar o efeito da fotobiomodulação combinada a resina autopolimerizável no controle da hipersensibilidade em dentes com HMI.

4.2 Objetivos Específicos

- Avaliar a longevidade das restaurações realizadas com uma resina composta autopolimerizável em primeiros molares acometidos por HMI.
- Comparar a eficácia de três intervenções (fotobiomodulação combinada à resina autopolimerizável, resina autopolimerizável isolada, e fotobiomodulação combinada à resina fotopolimerizável bulkfill) no controle da hipersensibilidade ao longo do tempo

5. Métodos

5.1 Delineamento do estudo

Será realizado um ensaio clínico controlado randomizado e cego. Por se tratar de um estudo clínico e buscando uma maior transparência e qualidade dessa pesquisa, utilizaremos as recomendações CONSORT (Consolidated Standards of ReportingTrials). (Figura 1)

Figura 1 Fluxograma CONSORT

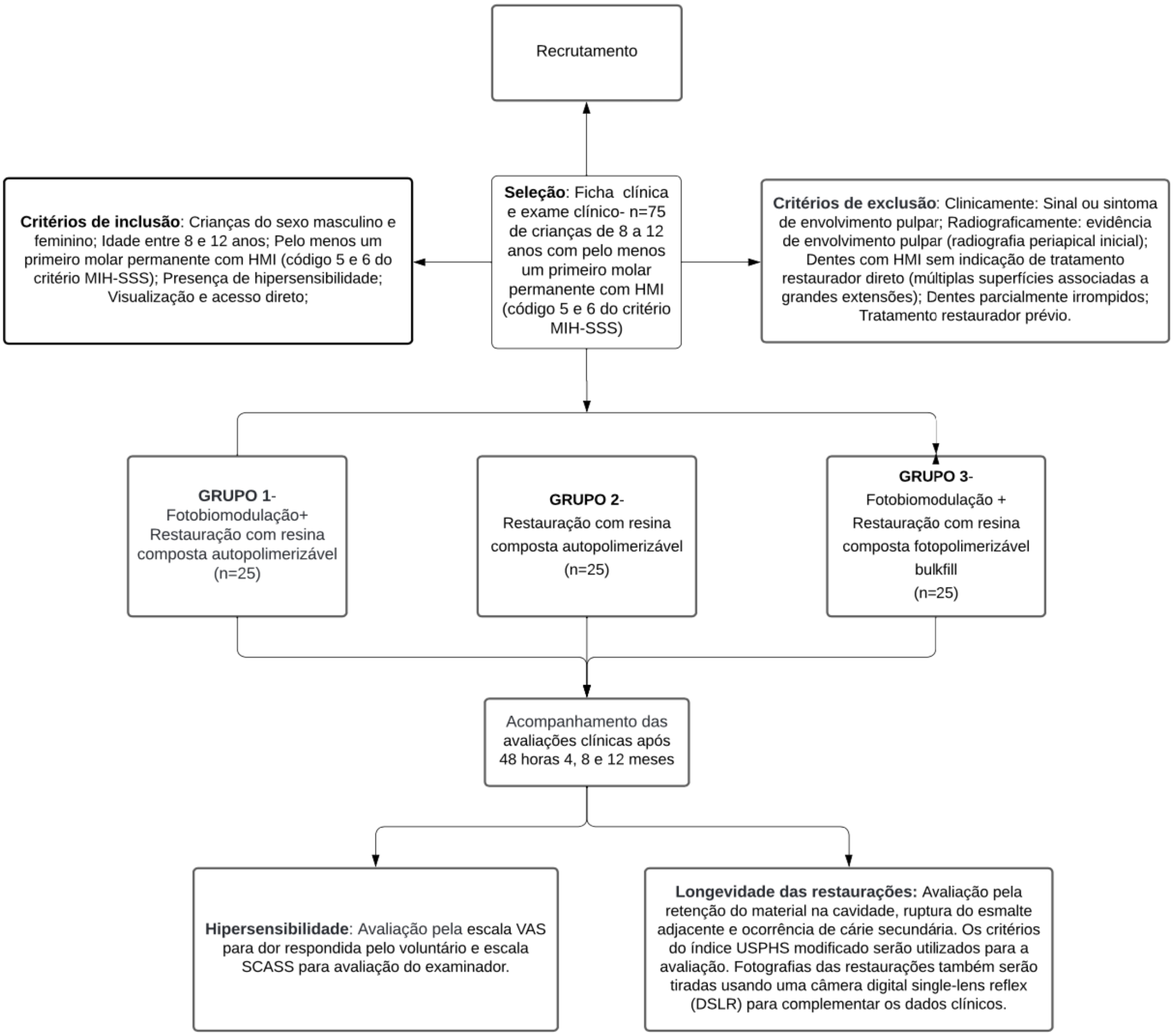

5.2 Aspectos éticos

O estudo será conduzido de acordo com os preceitos éticos estipulados na Declaração de Helsinque (Declaração da Associação Médica Mundial de Helsinque, 2008) e de acordo com as normas que regem pesquisas envolvendo seres humanos estipuladas nas Resoluções nº 466/12 e 510/2016 de do Conselho Nacional de Saúde. Os responsáveis legais concordarão com a participação das crianças assinando por escrito uma declaração de consentimento informado e as crianças através de um termo se

assentimento. O recrutamento será iniciado após aprovação do Comitê de Ética em Pesquisa com Seres Humanos.

Os participantes serão informados que poderão desistir do estudo a qualquer momento, por qualquer motivo, se assim o desejarem. Os pesquisadores também poderão retirar participantes do estudo se julgarem necessário.

5.3 Cálculo do tamanho da amostra

O tamanho da amostra será estimado usando o software G*Power (versão 3.1, Franz Faul, Unikiel, Alemanha) [21] para ANOVA de medidas repetidas a priori. Um tamanho de efeito de 0,352, derivado das dispersões médias do grupo relatadas na literatura [22], será considerado. O erro alfa será definido em 0,05, o poder estatístico em 80% e o delineamento inclui 3 grupos e 5 medidas repetidas. O cálculo final indica a necessidade de 66 indivíduos no total, correspondendo a 22 participantes por grupo. Para aumentar a robustez dos resultados e compensar potenciais desistências, a amostra será inflada em 15%, resultando em 25 indivíduos por grupo e um total de 75 participantes.

5.4 Descrição da amostra

Serão selecionados dentes de crianças do sexo masculino e feminino 8 até 12 anos de idade (sem distinção de etnia) cadastradas para tratamento no ambulatório do Instituto Aria em Brasília com primeiros molares apresentando HMI e necessidade de tratamento restaurador. Os indivíduos que atenderem aos critérios de inclusão serão imediatamente randomizados e tratados. Cada participante receberá tratamento de um único dente molar afetado pela HMI. Se mais de um dente atender aos critérios de inclusão, o dente a ser incluído no estudo será selecionado aleatoriamente por meio de sorteio simples. Caso o participante apresente mais de um dente afetado com HMI o/os outros dentes receberão o tratamento padrão da faculdade.

5.5 Critério de inclusão

- Crianças do sexo masculino e feminino.
- Idade entre 8 e 12 anos.
- Pelo menos um primeiro molar permanente com HMI (código 5 e 6 do critério MIH-SSS). [20]
- Presença de hipersensibilidade.
- Visualização e acesso direto.

5.6 Critério de exclusão

- Clinicamente: Sinal ou sintoma de envolvimento pulpar.
- Radiograficamente: evidência de envolvimento pulpar (radiografia periapical inicial).
- Dentes com HMI sem indicação de tratamento restaurador direto (múltiplas superfícies associadas a grandes extensões).
- Dentes parcialmente irrompidos.
- Tratamento restaurador prévio.

5.7 Envolvimento do Paciente e do Público

Os responsáveis pelos pacientes não estiveram envolvidos no desenho deste estudo. Após a análise dos dados, os responsáveis terão a oportunidade de participar de uma reunião de partilha de resultados, caso assim o desejem. O termo de consentimento assinado pelos responsáveis dos participantes explica que o armazenamento dos dados de cada participante e familiar ocorre dentro dos termos de confidencialidade.

5.8 **Randomização**

Os participantes serão alocados aleatoriamente em diferentes grupos usando um método de randomização em bloco. Uma sequência gerada por computador (random.org; Randomness and Integrity Services, Dublin, Leinster, Irlanda) será usada para alocar cada participante a um grupo específico mantendo uma proporção de 1:1:1 (número igual de participantes em todos os grupos). A ocultação da alocação será garantida com o uso de envelopes opacos lacrados e numerados sequencialmente. Os dentes selecionados para tratamento restaurador serão randomizados em três grupos:

Grupo 1: Fotobiomodulação + Restauração com resina composta autopolimerizável (NOVO)

Grupo 2: Restauração com resina composta autopolimerizável (NOVA)

Grupo 3: Fotobiomodulação + Restauração com resina composta fotopolimerizável bulkfill (PADRÃO OURO – MAIS UTILIZADA)

5.9 Cegamento

Todos os tratamentos serão realizados para os três grupos por um único operador que terá passado por exercícios de treinamento e calibração na fase inicial do estudo. Avaliações

clínicas após 48 horas, bem como aos 4, 8 e 12 meses de acompanhamento serão realizadas por um avaliador cego para os tratamentos (estudo simples-cego). O operador e o avaliador serão previamente calibrados (cálculo pela estatística Kappa) para garantir concordância inter-observadores superior a 85% com base nos critérios do índice modificado do Serviço de Saúde Pública dos Estados Unidos (USPHS) [22,23] bem como nos critérios de diagnóstico MIH- SSS [20]. Os dados serão analisados por um estatístico cego.

5.10 Intervenções

5.10.a Fotobiomodulação

O tratamento com a fotobiomodulação será realizado com laser infravermelho de diodo de baixa potência (DMC, São Carlos, Brasil) em única sessão. A aplicação será realizada em três pontos perpendiculares e em contato com a superfície, nos terços vestibular mesial e distal cervical, e no centro da face oclusal [24]. Cada ponto será submetido à aplicação por 10 segundos, com uma energia de 1 J. Os parâmetros estão descritos na tabela 1.

Tabela 1: Parâmetros – Fotobiomodulação	
Método de aplicação	Contato
Número de pontos irradiados	3
Número de sessões	1
Comprimento de onda (nm)	808 ± 10
Irradiância (mW/cm^2)	3571 mW/cm^2
Largura espectral (FWHM)	4,8 ± 2 nm
Regime de tempo	Contínuo
Potência	100 mW
Tipo de feixe	Multimodo
Área do feixe [cm^2]	0,028
Tempo de exposição total	30 s
Exposição radiante [J/cm^2]	35,7 J/cm^2
Energia por ponto [J]	1 J
Energia total [J]	3 J

5.10.b Protocolo clínico

Grupo 1- Fotobiomodulação + Restauração com resina composta autopolimerizável

1. Radiografia periapical inicial.
2. Tomada da hipersensibilidade (escala VAS para dor respondida pelo voluntário e escala SCASS para avaliação do examinador).
3. Aplicação da fotobiomodulação
4. Isolamento relativo (protetor labial, rolo de algodão e aspirador);
5. Remoção seletiva do tecido cariado com cureta de dentina (somente código 6 do critério MIH-SSS)
6. Limpeza com algodão e água;
7. Aplicação do primer (Stela; SDI, Melbourne, Vic, Austrália) em dentina e/ou esmalte, aguardar 5 segundos;
8. Aplicação de ar comprimido suave sobre o adesivo por 3 segundos;
9. Restauração com resina composta autopolimerizável (Stela; SDI, Melbourne, Vic, Austrália) estendendo-se até opacidades demarcadas adjacentes;
10. Acompanhamento clínico após 48 horas e em intervalos de quatro meses por um período de 12 meses (índice USPHS modificado);
11. Tomada da hipersensibilidade após 48 horas e em intervalos de quatro meses por um período de 12 meses (escala VAS para dor respondida pelo voluntário e escala SCASS para avaliação do examinador).

Grupo 2- Restauração com resina composta autopolimerizável

A mesma sequência anterior (grupo 1) com exceção do item 3.

Grupo 3 – Fotobiomodulação + Restauração com resina composta bulkfill

Grupo tratamento padrão para a condição de saúde bucal descrita.

1. Radiografia periapical inicial.
2. Tomada da hipersensibilidade (escala VAS para dor respondida pelo voluntário e escala SCASS para avaliação do examinador).
3. Isolamento relativo (protetor labial, rolo de algodão e aspirador);
4. Aplicação da fotobiomodulação;
5. Remoção seletiva do tecido cariado com cureta de dentina (somente código 6 do critério MIH-SSS)

6. Condicionamento seletivo do esmalte adjacente e opacidades demarcadas com ácido fosfórico 35% (Ultra Etch; Ultradent, Indaiatuba, S, Brasil) por 20 segundos;
7. Aplicação do adesivo universal (Ambar; FGM, Joinville, SC, Brasil) em dentina e esmalte de forma ativa por 20 segundos (repetir procedimento);
8. Leve jato de ar sobre o adesivo por 5 segundos;
9. Fotoativação por 10 segundos 1200Mw/cm^2 pico (Radii Cal; SDI, Melbourne, Vic, Austrália);
10. Restauração com resina composta bulkfill Tetric N Ceram (Tetric N Ceram Bulk Fill; Ivoclar Vivadent, Barueri, SP, Brasil) com incrementos de até 4mm estendendo para opacidades demarcadas;
11. Fotoativação por 10 segundos (Radii Cal; SDI, Melbourne, Vic, Austrália);
12. Acompanhamento clínico após 48 horas e em intervalos de quatro meses por um período de 12 meses (índice USPHS modificado);
13. Tomada da hipersensibilidade após 48 horas e em intervalos de quatro meses por um período de 12 meses (escala VAS para dor respondida pelo voluntário e escala SCASS para avaliação do examinador).

Todas as intervenções serão iniciadas sem a administração prévia de anestesia local. Cada criança será informada que a anestesia pode ser administrada a qualquer momento durante a intervenção.

6. Avaliações

6.1 Avaliações radiográficas

Radiografias periapicais serão realizadas para descartar a possibilidade de envolvimento pulpar. Radiografias de acompanhamento só serão realizadas se os sintomas de dor justificarem a exposição.

6.2 Avaliação Clínica

A avaliação clínica será realizada por um avaliador que não terá conhecimento do grupo de tratamento ao qual cada dente está alocado. Os critérios utilizados para a avaliação clínica serão a retenção do material restaurador na cavidade, ruptura do esmalte adjacente à restauração e ocorrência de cárie secundária. Os critérios do índice USPHS modificado serão utilizados para a avaliação. A restauração será caracterizada como falha e o dente

será excluído do estudo se o escore C for determinado para qualquer um dos critérios do USPHS [25]. Fotografias das restaurações também serão tiradas usando uma câmera digital single-lens reflex (DSLR) (Canon EOS 700D; Canon, Tóquio, Honshu, Japão) para complementar os dados clínicos. A demonstração visual poderá contribuir para eventuais esclarecimentos necessários, bem como tornar mais eficiente a discussão e documentação dos casos.

6.3 Avaliação de Hipersensibilidade

Será utilizada a Escala Analógica Visual (Visual Analogue Scale - VAS) [26] e escala Schiff para sensibilidade ao ar frio (SCASS) para avaliação do operador e examinador que seguirá o seguinte protocolo: isolamento com gaze dos dentes vizinhos e jato de ar no dente com HMI durante 3 segundos. A escala SCASS apresenta 4 pontuações: 0 (não reage); 1 (não reage, mas considera o desconforto); 2 (reage e se movimenta); 3 (reage e pede para parar) [3]. A tomada da hipersensibilidade através da escala VAS e SCASS será realizada antes do procedimento e repetida 48 horas apósa o tratamento restaurador e a cada 4 meses por um período de 12 meses de acompanhamento.

7. Análise Estatística

A normalidade da distribuição dos dados e a homogeneidade da variância serão confirmadas pelos testes de Shapiro-Wilk e Levene, respectivamente. Os dados serão apresentados por meio de estatística descritiva. As variáveis contínuas serão descritas com média e desvio padrão, e as variáveis categóricas por frequência relativa. As análises estatísticas serão conduzidas usando o software SPSS (versão 28.0, IBM, EUA). Para comparações das escalas VAS e SCASS, será usada uma análise de variância de medidas repetidas (ANOVA), considerando os três grupos e cinco momentos do estudo. As comparações post-hoc serão feitas usando o ajuste de Bonferroni para controlar o erro Tipo I em comparações múltiplas. A presença de esfericidade será verificada usando o teste de Mauchly e, se violada, serão aplicadas correções como Greenhouse-Geisser. Um nível de significância de 0,05 será adotado para todas as análises.

CRONOGRAMA GERAL

O cronograma previsto para a pesquisa será executado caso o projeto seja APROVADO pelo Sistema CEP/CONEP". Os procedimentos terão início somente após a aprovação do comitê de ética em pesquisas em seres humanos.

**Identificação da Etapa**	**Início (25/11/2024)**	**Término (11/08/2026)**
Enviar para o Comitê de Ética	25/11/2024	25/11/24
Convite e recrutamento (após aprovação do CEP)	01/02/2025	15/02/2025
Coleta de dados (após aprovação do CEP)	16/02/2025	01/01/2026
Tabulação dos dados (após aprovação do CEP)	02/01/2026	01/03/2026
Análise dos dados	02/03/2026	02/04/2026
Redação dos resultados	03/04/2026	01/08/2026

Previsão Orçamentária

Material de consumo/ equipamento	Quantidade	Valor Unitário	Previsão orçamentária	Existente	Necessária aquisição
Resina composta autopolimerizável Stela + Stela Primer	50(1 caixa com 10 cápsulas)	R$ 386,00 (caixa com 10 cápsulas)	R$ 1930,00	( ) Sim<br>(x) Não	(x) Sim<br>( ) Não
Aplicador para inserção na cavidade	1	R$ 358,00	R$ 358,00	(x) Sim<br>( ) Não	( ) Sim<br>(x) Não
Amalgamador Ultramat S - Bivolt - SDI	1	R$2.883,90	R$2.883,90	(x) Sim<br>( ) Não	( ) Sim<br>(x) Não
Filme Radiográfico Adulto Periapical E-Speed - Carestream	100	R$208,99 (caixa com 150 unidades)	R$208,99	( ) Sim<br>(x) Não	(x) Sim<br>( ) Não
Resina composta fotopolimerizável bulkfill SDI	4	R$182,90	R$731,60	( ) Sim<br>(x) Não	(x) Sim<br>( ) Não
Fotopolimerizador Valo Cordless Grand - Ultradent	1	R$9.898,99	R$9.898,99	(x) Sim<br>( ) Não	( ) Sim<br>(x) Não
Papacárie	2	R$149,90	R$299,80	( ) Sim<br>(x) Não	(x) Sim<br>( ) Não
Laser Therapy XT (DMC)	1	R$5.175,10	R$5.175,10	(x) Sim<br>( ) Não	( ) Sim<br>(x) Não
Gorro descartável	2 embalagens	R$ 33,82 (embalagem com 100 unidades)	R$ 67,64	( ) Sim<br>(x) Não	(x) Sim<br>( ) Não
Máscara descartável	2 caixas	R$ 29,90 (caixa com 50 unidades)	R$ 59,80	( ) Sim<br>(x) Não	(x) Sim<br>( ) Não
Luva de procedimento	10 caixas	R$ 25,00 (caixa com 50 unidades)	R$ 250,00	( ) Sim<br>(x) Não	(x) Sim<br>( ) Não
Avental descartável	7 pacotes	R$ 53,80 (pacote com 10 unidades)	R$ 376,60	( ) Sim<br>(x) Não	(x) Sim<br>( ) Não
Sugador descartável	6 pacotes com 40 unidades	R$ 7,00 (com 40 unidades)	R$ 42,00	( ) Sim<br>(x) Não	(x) Sim<br>( ) Não

Rolete de algodão	2 pacote com 500 unidades	R$ 79,87 (com 500 unidades)	R$ 159,74	( ) Sim<br>(x) Não	(x) Sim<br>( ) Não

Os custos do projeto serão arcados pelo pesquisador.

Referências:

1. Bussaneli DG, Vieira AR, Santos-Pinto L, Restrepo M. Molar-incisor hypomineralisation: an updated view for aetiology 20 years later. Eur Arch Paediatr Dent. 2022 Feb;23(1):193-198. doi: 10.1007/s40368-021-00659-6. Epub 2021 Aug 15. PMID: 34392496.
2. Negre-Barber A, Montiel-Company JM, Catalá-Pizarro M, Almerich-Silla JM. Degree of severity of molar incisor hypomineralization and its relation to dental caries. Sci Rep. 2018 Jan 19;8(1):1248. doi: 10.1038/s41598-018-19821-0. PMID: 29352193.
3. Linner T, Khazaei Y, Bücher K, Pfisterer J, Hickel R, Kühnisch J. Hypersensitivity in teeth affected by molar-incisor hypomineralization (MIH). Sci Rep. 2021 Sep 9;11(1):17922. doi: 10.1038/s41598-021-95875-x. PMID: 34504122; PMCID: PMC8429747.
4. Raposo F, de Carvalho Rodrigues AC, Lia ÉN, Leal SC. Prevalence of Hypersensitivity in Teeth Affected by Molar-Incisor Hypomineralization (MIH). Caries Res. 2019;53(4):424-430. doi: 10.1159/000495848. Epub 2019 Jan 24. PMID: 30677762.
5. Yonaga K, Kimura Y, Matsumoto K. Treatment of cervical dentin hypersensitivity by various methods using pulsed Nd:YAG laser. J Clin Laser Med Surg. 1999 Oct;17(5):205-10. doi: 10.1089/clm.1999.17.205. PMID: 11199824.
6. Brännström M, Johnson G, Nordenvall KJ. Transmission and control of Dentinal pain: resin Impregnation for the desensitization of Dentin. *J Am Dent Assoc* 1979;99:612–8. 10.14219/jada.archive.1979.0337
7. Machado AC, Viana ÍEL, Farias-Neto AM, et al.. Is Photobiomodulation (PBM) effective for the treatment of Dentin hypersensitivity? A systematic review. *Lasers Med Sci* 2018;33:745–53. 10.1007/s10103-017-2403-7
8. Lagarde M, Vennat E, Attal JP, Dursun E. Strategies to optimize bonding of adhesive materials to molar-incisor hypomineralization-affected enamel: A systematic review. Int J Paediatr Dent. 2020 Jul;30(4):405-420. doi: 10.1111/ipd.12621. Epub 2020 Feb 12. PMID: 31990108.
9. Ekambaram M, Anthonappa RP, Govindool SR, Yiu CKY. Comparison of deproteinization agents on bonding to developmentally hypomineralized enamel. J Dent.

2017 Dec;67:94-101. doi: 10.1016/j.jdent.2017.10.004. Epub 2017 Oct 12. PMID: 29031995.

10. Grossi JA, Cabral RN, Ribeiro APD, Leal SC. Glass hybrid restorations as an alternative for restoring hypomineralized molars in the ART model. BMC Oral Health. 2018 Apr 18;18(1):65. doi: 10.1186/s12903-018-0528-0. PMID: 29669561
11. Raskin A, Setcos JC, Vreven J, Wilson NH. Influence of the isolation method on the 10-year clinical behaviour of posterior resin composite restorations. Clin Oral Investig. 2000 Sep;4(3):148-52. doi: 10.1007/s007840000069. PMID: 11000319.
12. Miao C, Yang X, Wong MC, Zou J, Zhou X, Li C, Wang Y. Rubber dam isolation for restorative treatment in dental patients. Cochrane Database Syst Rev. 2021 May 17;5(5):CD009858. doi: 10.1002/14651858.CD009858.pub3. PMID: 33998662
13. Sabbagh J, Dagher S, El Osta N, Souhaid P. Randomized Clinical Trial of a Self-Adhering Flowable Composite for Class I Restorations: 2-Year Results. Int J Dent. 2017;2017:5041529. doi: 10.1155/2017/5041529. Epub 2017 Mar 1. PMID: 28348594
14. Andrea Kowalska et al. The Photoinitiators Used in Resin Based Dental Composite – A Review and Future Perspectives, 2021
15. Andrea Kowalska et al. Can TPO as Photoinitiator Replace “Golden Mean” Camphorquinone and Tertiary Amines in Dental Composites?,2022
16. Hamdi Hosni Hamama. Recent advances in posterior resin composite restorations in Applications of Nanocomposite Materials in Dentistry, 2019.
17. S.R. Schricker. Composite resin polymerization and relevant parameters in Orthodontic Applications of Biomaterials, 2017.
18. Gary S. Berkowitz et al. Postoperative Hypersensitivity and Its Relationship to Preparation Variables in Class I Resin-Based Composite Restorations: Findings from the Practitioners Engaged in Applied Research and Learning (PEARL) Network. Part 1. Compend Contin Educ Dent. 2013 Mar; 34(3): e44–e52.
19. Salvatore Sauro et al.Microtensile bond strength and interfacial adaptation of two bulk-fill composites compared to a conventional composite restorative system, 2022.
20. Cabral RN, Nyvad B, Soviero VLVM, Freitas E, Leal SC. Reliability and validity of a new classification of MIH based on severity. Clin Oral Investig. 2020 Feb;24(2):727-734. doi: 10.1007/s00784-019-02955-4. Epub 2019 May 25. PMID: 31129878.
21. Bardellini, E., Amadori, F., Rosselli, L., Garo, M. L., Majorana, A., & Conti, G. (2024). Molar Incisor Hypomineralization: Optimizing Treatment Protocols for Hypersensitivity: A Randomized Clinical Trial. *Dentistry journal*, *12*(6), 186. https://doi.org/10.3390/dj12060186
22. Rolim TZC, da Costa TRF, Wambier LM, Chibinski AC, Wambier DS, da Silva Assunção LR, de Menezes JVBN, Feltrin-Souza J. Adhesive restoration of molars

affected by molar incisor hypomineralization: a randomized clinical trial. Clin Oral Investig. 2021 Mar;25(3):1513-1524. doi: 10.1007/s00784-020-03459-2. Epub 2020 Jul 21. PMID: 32696210.

23. Sönmez H, Saat S. A Clinical Evaluation of Deproteinization and Different Cavity Designs on Resin Restoration Performance in MIH-Affected Molars: Two-Year Results. J Clin Pediatr Dent. 2017;41(5):336-342. doi: 10.17796/1053-4628-41.5.336. PMID: 28872993.
24. Fossati AL, Sobral APT, Hermida Bruno MLL, Viarengo NO, Sertaje MRF, Santos EM, Gonçalves MLL, Ferrari RAM, Fernandes KPS, Horliana ACRT, Motta LJ, Bussadori SK. Photobiomodulation and glass ionomer sealant as complementary treatment for hypersensitivity in molar incisor hypomineralisation in children: protocol for a blinded randomised clinical trial. BMJ Open. 2023 Jun 14;13(6):e068102. doi: 10.1136/bmjopen-2022-068102. PMID: 37316315; PMCID: PMC10277091.
25. de Souza JF, Fragelli CB, Jeremias F, Paschoal MAB, Santos-Pinto L, de Cássia Loiola Cordeiro R. Eighteen-month clinical performance of composite resin restorations with two different adhesive systems for molars affected by molar incisor hypomineralization. Clin Oral Investig. 2017 Jun;21(5):1725-1733. doi: 10.1007/s00784-016-1968-z. Epub 2016 Oct 15. PMID: 27743215.
26. Jensen MP, Karoly P, Braver S. The measurement of clinical pain intensity: a comparison of six methods. Pain. 1986 Oct;27(1):117-126. doi: 10.1016/0304-3959(86)90228-9. PMID: 3785962.
27. Americano GC, Jacobsen PE, Soviero VM, Haubek D. Uma revisão sistemática sobre a associação entre hipomineralização de incisivos molares e cárie dentária. *Int. J. Pediatra. Dente.* 2017; **27** :11-21. doi: 10.1111/ipd.12233.
28. Jälevik B, Klingberg GA. Dental treatment, dental fear and behaviour management problems in children with severe enamel hypomineralization of their permanent first molars. Int J Paediatr Dent. 2002 Jan;12(1):24-32. PMID: 11853245.
